# An Overview of *Monkeypox Virus* Detection in Different Clinical Samples and Analysis of Temporal Viral Load Dynamics

**DOI:** 10.1002/jmv.70104

**Published:** 2024-12-09

**Authors:** Rita Cordeiro, Ana Pelerito, Isabel Lopes de Carvalho, Sílvia Lopo, Raquel Neves, Raquel Rocha, Paula Palminha, Nuno Verdasca, Cláudia Palhinhas, Maria José Borrego, Carla Manita, Idalina Ferreira, Célia Bettencourt, Patrícia Vieira, Sónia Silva, Ivone Água‐Doce, Carla Roque, Dora Cordeiro, Greice Brondani, João Almeida Santos, Susana Martins, Irene Rodrigues, Carlos Ribeiro, Maria Sofia Núncio, João Paulo Gomes, Fernando da Conceição Batista

**Affiliations:** ^1^ Emergency Response and Biopreparedness Unit, Infectious Diseases Department National Institute of Health Doutor Ricardo Jorge Lisbon Portugal; ^2^ Institute of Environmental Health, Faculty of Medicine University of Lisbon Lisbon Portugal; ^3^ National Reference Laboratory for Sexually Transmitted Infections, Infectious Diseases Department National Institute of Health Doutor Ricardo Jorge Lisbon Portugal; ^4^ National Reference Laboratory for Vaccine‐Preventable Diseases, Infectious Diseases Department National Institute of Health Doutor Ricardo Jorge Lisbon Portugal; ^5^ Management Support Unit, Infectious Diseases Department National Institute of Health Doutor Ricardo Jorge Lisbon Portugal; ^6^ Genomics and Bioinformatics Unit National Institute of Health Doutor Ricardo Jorge (INSA) Lisbon Portugal; ^7^ Veterinary and Animal Research Center (CECAV), Faculty of Veterinary Medicine Lusófona University Lisbon Portugal; ^8^ School of Technology and Management, Centre for Rapid and Sustainable Product Development Polytechnic Institute of Leiria Leiria Portugal

**Keywords:** clinical samples, *C*_t_ values, *Monkeypox virus*, Portugal, positive rate, viral clearance, viral load

## Abstract

Mpox is a zoonotic disease caused by the *Monkeypox virus* (MPXV), and since May 2022, tens of thousands of cases have been reported in non‐endemic countries. We aimed to evaluate the suitability of different sample types for mpox diagnostic and assess the temporal dynamics of viral load. We evaluated 1914 samples from 953 laboratory‐confirmed cases. The positivity rate was higher for lesion (91.3%) and rectal swabs (86.1%) when compared with oropharyngeal swabs (69.5%) and urines (41.2%), indicating higher viral loads for the former. Supporting this, lesion and rectal swabs showed lower median PCR *C*_t_ values (*C*_t_ = 23 and *C*_t_ = 24), compared to oropharyngeal swabs and urines (*C*_t_ = 31). Stable MPXV loads were observed in swabs from lesions up to 30 days after symptoms onset, contrasting with a considerable decrease in viral load in rectal and oropharyngeal swabs. Overall, these results point to lesion swabs as the most suitable samples for detecting MPXV in the 2022–2023 multicountry outbreak and show comparable accuracy to rectal swabs up to 8 days after symptoms onset. These findings, together with the observation that about 5% of patients were diagnosed through oropharyngeal swabs while having negative lesions, suggest that multisite testing should be performed to increase diagnostic sensitivity.

## 1 Introduction

*Monkeypox virus* (MPXV) belongs to the *Poxviridae* family and *Orthopoxvirus* genus, which also includes the smallpox virus. MPXV was discovered in Denmark in 1958 in monkeys in a research laboratory, and the first human disease case was reported in 1970 in a 9‐month‐old boy in the Democratic Republic of Congo, Central Africa. Mpox is a rare and endemic zoonotic disease in West and Central African countries, where close interaction between humans and wild animals or reservoir(s) is common. MPXV is known to be spread through close contact with lesions, body fluids and respiratory droplets of infected humans or animals [1, 2].

MPXV genetic diversity involves the classification into two clades: clade I (formerly designated Congo Basin clade), enrolling subclades Ia and Ib, and clade II (formerly designated West African clade), enrolling subclades IIa and IIb. Clade I is generally associated with higher virulence and severity when compared with clade II [3, 4, 5].

Since the beginning of May 2022, several cases of MPXV subclade IIb have been reported in numerous countries where the disease is not endemic, affecting primarily men who have sex with men (MSM). Until September 2024, almost 100 000 confirmed cases have been reported by the World Health Organization (WHO), in more than 100 countries [3].

The WHO declared the mpox outbreak a Public Health Emergency of International Concern on July 23, 2022, reinforcing the importance of maintaining epidemiological surveillance of the disease. The International Health Regulations Emergency Committee declared the emergency status terminated on May 10, 2023. Currently, the WHO continues to consider that the global risk is moderate, including in the European Region [4].

On August 14, 2024, triggered by the considerable increase in the number of MPXV clade Ib infections in the Democratic Republic of the Congo, accompanied by the geographical spread of the virus to neighbouring countries, the WHO declared again mpox as a Public Health Emergency of International Concern. Meanwhile, MPXV clade Ib cases outside Africa, namely in Sweden, Germany, Thailand, India, and the United Kingdom, were reported in individuals with a history of travel to regions in Africa where the virus is actively circulating [3].

The typical presentation of mpox consists of a short prodromal febrile period followed by the progressive development of a classic skin rash with hardened and umbilicated lesions, starting on the head or face and progressing to the limbs and trunk, all at the same stage. Lymphadenopathy is typical of this disease. However, in the recent outbreak, there were reports that the progression of the lesions may be atypical, and begin in the genital area. The clinical differential diagnosis must consider other exanthematous diseases, particularly chickenpox [5].

Laboratory confirmation of mpox is preferably carried out using nucleic acid amplification tests (NAAT), such as real time or conventional polymerase chain reaction (PCR). The NAAT can be generic for *Orthopoxvirus* or, preferably, specific for MPXV. Real‐time PCR is the preferred technique for laboratory diagnosis given its precision and sensitivity associated with obtaining results quickly and robustly, essential characteristics in the initial investigation of cases. In addition to NAAT, viral genome sequencing is useful for determining virus clade and understanding transmission dynamics and epidemiology [6].

Traditionally, the appropriate sample for laboratory confirmation of mpox is the lesion swab, but the collection of an oropharyngeal swab is also recommended. However, considering the sexual contact nature of most confirmed transmissions within the current global outbreak, depending on the patient's clinical manifestations, the collection of genital and/or rectal swabs, urine and semen might be considered [6].

Rapid and early detection, as well as the implementation of prevention and control measures, are essential, which highlights the importance of reference laboratories with the capacity to support emergency response measures and contingency plans.

Since 2007, the Portuguese National Institute of Health Doutor Ricardo Jorge (INSA) has a Unit specialized in Emergency Response and Biopreparedness (UREB). UREB, which is part of the Department of Infectious Diseases, is the national reference laboratory for the detection of highly pathogenic agents with pandemic potential, including *Orthopoxvirus*.

Portugal was one of the first countries to report cases of mpox, with the first case reported on May 17, 2022 [7]. The last confirmed positive case of the first wave was detected in April 2023. In June 2023, a new wave of the outbreak started, with 229 confirmed cases identified until March 2024.

The present study aims to describe MPXV detection in different clinical samples tested in the Portuguese reference laboratory, and to evaluate the accuracy of each sample type for this specific diagnostics.

## 2 Methods

### 2.1 Laboratory Procedures

Clinical samples were collected from patients suspected of having mpox disease based on clinical observation, in around 100 governmental and nongovernmental healthcare facilities throughout the country. All samples were received by UREB at INSA for MPXV screening.

A total of 4125 samples of different specimen types from 2016 patients were processed, namely: lesion swabs (*n* = 2142/4125, 51.9%), oropharyngeal swabs (*n* = 1703/4125, 41.3%), rectal swabs (*n* = 162/4125, 3.9%), urine samples (*n* = 70/4125, 1.7%), and others (*n* = 48/4125, 1.2%) that included genital and ocular swabs, semen, cerebrospinal fluid, peripheral blood, pleural fluid, serum, and bronchial secretions. As expected, the number of lesion and oropharyngeal swabs tested was the highest as these were the recommended samples by the WHO for mpox diagnosis [6]. Nevertheless, as the outbreak progressed, the laboratory also requested other types of samples, such as rectal swabs and urine, which were also used for diagnostic purposes.

DNA extraction from clinical samples was performed using the MagMAX Viral/Pathogen Nucleic Acid Isolation kit in a KingFisher Extractor (ThermoFisher), according to the manufacturer's recommendations.

Diagnosis was based on an in‐house real‐time PCR MPXV virus‐specific identification (B7R gene) protocol, as previously described [8]. Positive, negative and internal (RNAseP gene) controls were included in all runs. All assays were performed on CFX Opus Real‐Time PCR System (Bio‐Rad). MPXV detection was categorized according to obtained PCR cycle threshold (Ct) values as negative (*C*_t_ ≥ 40), weakly positive (35 ≤ *C*_t_ < 40), or positive (*C*_t_ < 35). The laboratory confirmation of mpox disease relied on the existence of at least one real‐time PCR‐positive sample per patient.

### 2.2 Statistical Analysis

Analyses and data visualization were performed using GraphPad Prism (version 8.3.0), and two‐sided *p* < 0.05 was considered statistically significant.

Comparisons of sample features between positive and negative groups were performed using chi‐square/Fisher's exact tests for categorical variables. Kruskal–Wallis tests were used to compare *C*_t_ values between four different types of clinical samples. For paired samples that passed the normality test, the *t*‐test was used while for others, the Wilcoxon test was used.

### 2.3 Regression Models

To assess time to viral clearance, a commutative analysis of positive samples with the Farazdaghi‐Harris density function ($D\left(t\right)={\left(a+b∙t^{c}\right)}^{-1}$), which uses three parameters, was performed [9].

To analyze the viral load increase in the first few days of symptoms and the slow decrease over the following weeks, an adaptation of the Gompertz growth curve (Ct(t)=40−a∙b∙exp(−a∙(t−c)−exp(−a∙(t−c))) was used. This curve also has three parameters to describe this temporal evolution [10].

### 2.4 Ethics Considerations

This research complies with all relevant ethical regulations. INSA is the national reference laboratory, being the Portuguese laboratory authorized by the General Directorate of Health (through the Technical Orientation N° 004/2022 of May 31, 2022) to process samples for MPXV detection and genetic characterization. Furthermore, this study essentially aimed at comparing the suitability of different sample types for diagnostics and assess viral load dynamics throughout infection, so samples were processed in an anonymized fashion, and no patient metadata that could identify patients was accessed or used.

## 3 Results

### 3.1 Mpox Positive Cases

**Figure 1:**
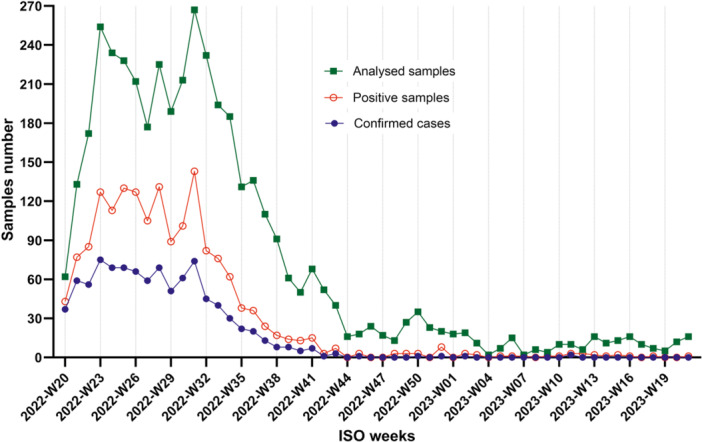
Distribution of the number of samples analyzed and confirmed cases between May 17, 2022, and May 24, 2023.

Between May 17, 2022, and May 24, 2023, 953 mpox cases were laboratory‐confirmed and reported in Portugal, having the prevalence peak in ISO week 23 of June 2022 (Figure 1 and Table S1). The majority of patients were male (*n* = 944; 99.1%), in the 30–39 age group (*n* = 420; 44.1%), with ages ranging from 17 to 66 years old (median = 34 years), and were mostly MSM. There were only nine cases (*n* = 9; 0.9%) in females, two of them were pregnant women, and also mostly in the 30–39 age group (*n* = 7; 77.8%) (Table S2). One case was a healthcare professional [11], and only one death was reported [12].

Additionally, a retrospective study was carried out with 84 genital ulcer swabs from 81 patients, collected in clinics located in the Lisbon Metropolitan Area and that had been sent to the reference laboratory for Sexually Transmitted Infections, between January 3 and May 17, 2022. Two samples from April 2022 tested positive for MPXV, indicating that the virus was already circulating in Portugal the first cases were notified.

Positive cases were detected in all regions of the country, but it was in the Lisbon Metropolitan Area that the highest number was recorded (*n* = 742/953; 77.9%), followed by the North Region (*n* = 158/953; 16.6%) and Central Region (*n* = 27/953; 2.8%) (Table S3).

Although most of the cases with known information were of foreign origin, 69.6% (*n* = 732/849) resided in Portugal at the time of diagnosis, 547 in the Lisbon Metropolitan Area and 114 in the North Region [13].

### 3.2 Assessment of MPXV PCR Positivity in Different Sample Types

**Figure 2:**
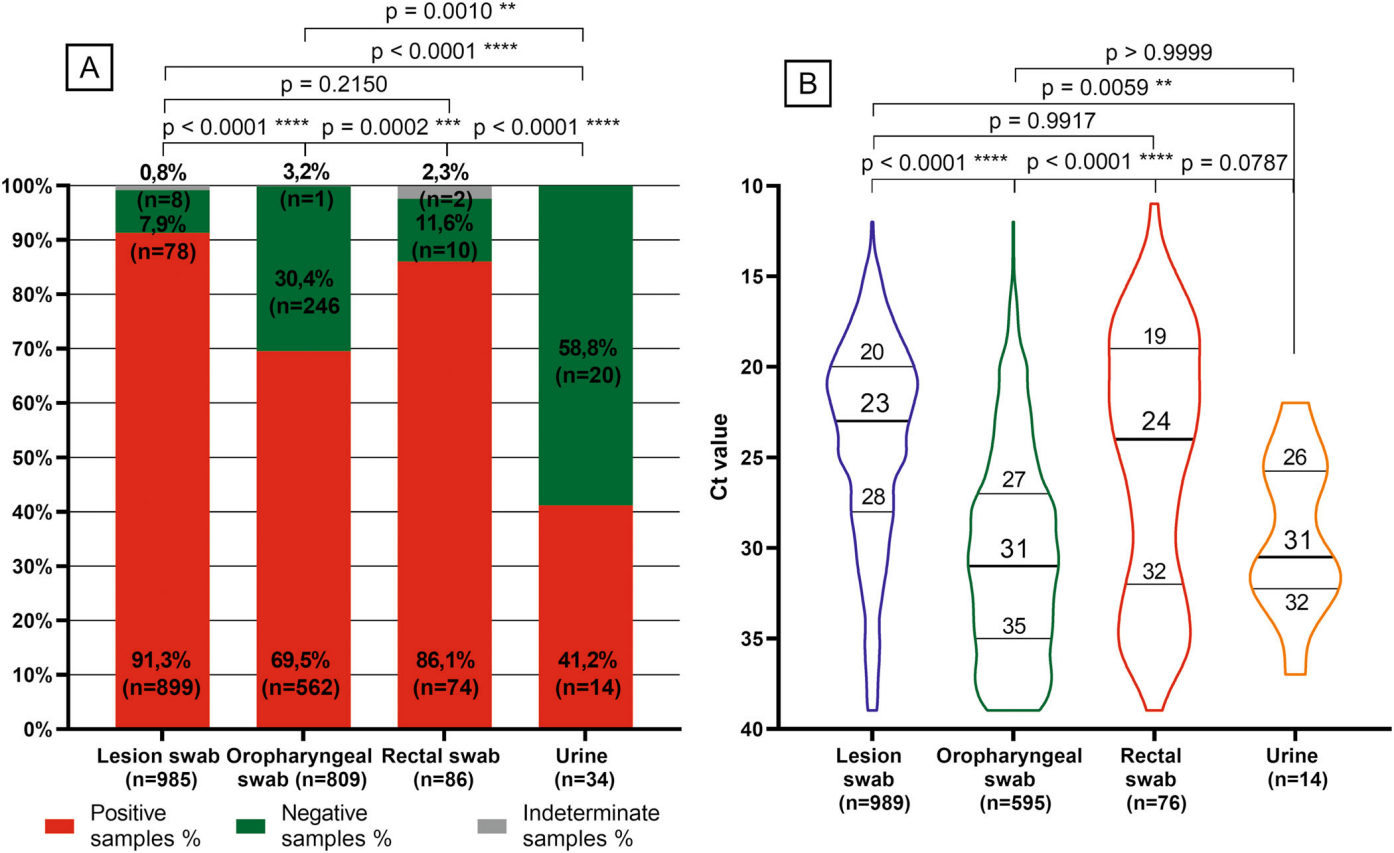
(A) Positive rate of MPXV detection according to the type of samples from the 953 confirmed cases. (B) MPXV viral loads are given as *C*_t_ values, according to the type of samples. For this analysis, follow‐up positive samples were also considered. Results are presented as Violin plots, with median and interquartile ranges represented. The *p* values refer to the comparison between being MPXV positive or negative by type of sample. The indeterminate sample number was not considered. *p* values are accompanied by asterisks, depending on the degree of significance (i.e., from one asterisk for *p* values less than 0.05 to four asterisks for *p* values less than 0.0001).

MPXV was detected in 1702 samples from 953 patients. To access the accuracy of different sample types to diagnose mpox, all samples (i.e., positive and negative, *n* = 1914) collected from the 953 positive patients were considered. The positivity rate was higher for lesion swabs (*n* = 899/985; 91.3%) and rectal swabs (*n* = 74/86; 86.1%) when compared with oropharyngeal swabs (*n* = 562/809; 69.5%) and urines (*n* = 14/34; 41.2%). A significant difference in positivity rate was verified between the different types of sample, except when comparing lesion and rectal swabs (Figure 2A). Nevertheless, some patients had undetectable MPXV from lesion swabs and were diagnosed with a positive oropharyngeal swab (*n* = 38/953; 4.8%), rectal swab (*n* = 5/953; 0.5%) or both (*n* = 6/953; 0.6%), which were taken in the same day as the corresponding lesion sample. These patients were tested either because they were contacts of MPXV‐positive patients or due to risk behaviours.

### 3.3 MPXV Viral Loads (*C*_t_ Values) and Time to Viral Clearance

Lesion and rectal swabs showed lower median values of *C*_t_ of 23 (interquartile range [IQR] 20–28) and 24 (IQR 19–32), respectively, suggestive of higher viral loads, compared to oropharyngeal swabs (*C*_t_ = 31, IQR 27–35) and urines (*C*_t_ = 31, IQR 26–32) (Figure 2B). These differences in viral loads were statistically different (Figure 2B).

A total of 1020 samples, with available information regarding the time from symptoms onset, were analysed, including lesion swabs (*n* = 611), oropharyngeal swabs (*n* = 352), rectal swabs (*n* = 47), and urines (*n* = 10).

**Figure 3:**
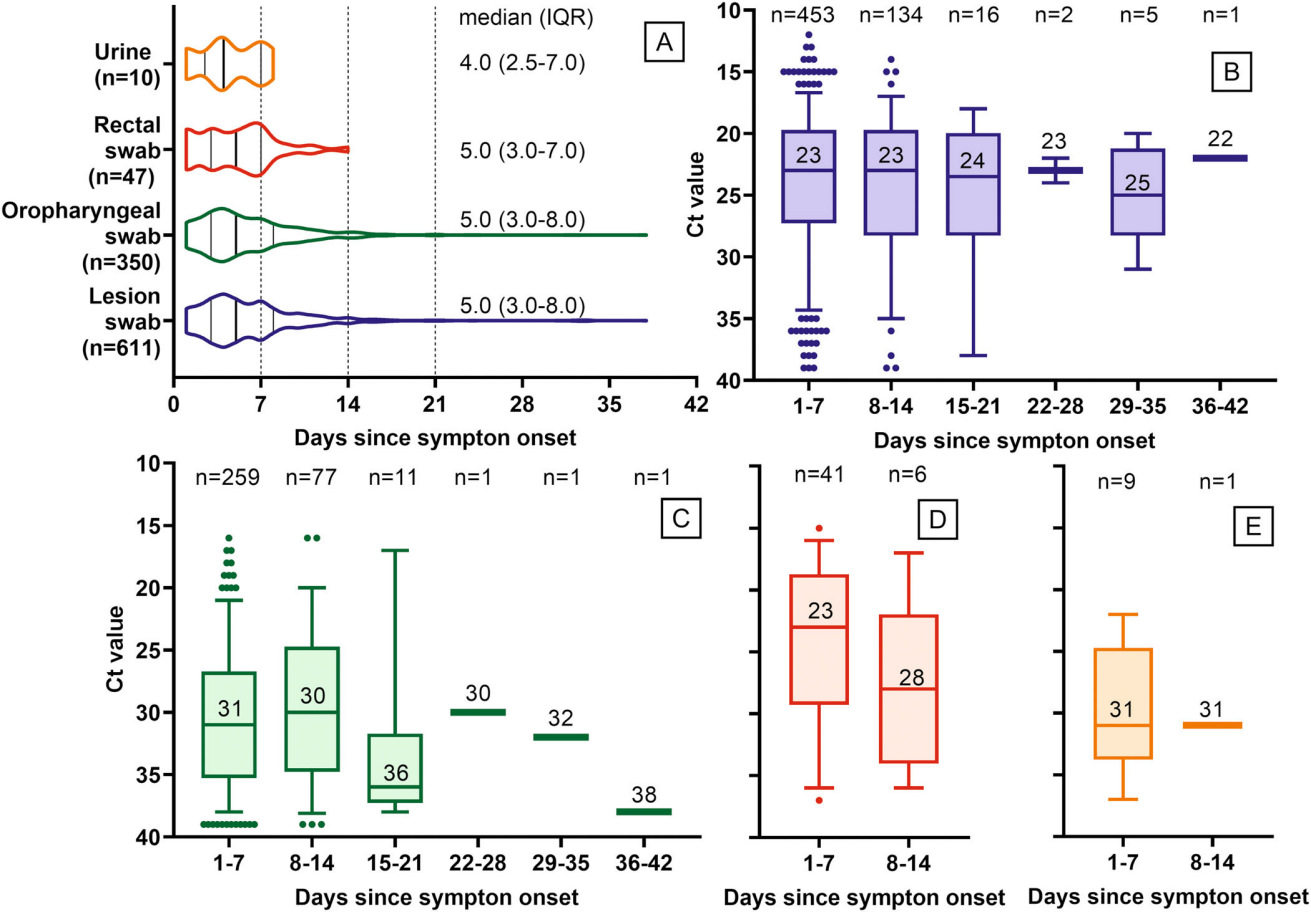
Median time from symptoms onset until MPXV diagnostic according to sample type (A) and PCR *C*_t_ values (B–E). (B–E) Lesion swabs (*n* = 611), oropharyngeal swabs (*n* = 352), rectal swabs (*n* = 47), and urine (*n* = 10), respectively. Results are presented as box and whisker plots, with median and interquartile ranges represented in boxes, and range presented as whiskers. Dots at the end of the whiskers represent 5% of the data. *C*_t_ values are represented in the axis in decreasing order for better interpretation purposes.

The median time from symptoms onset until MPXV diagnostic was 5 days for lesions (IQR 2.5–7.0 days), oropharyngeal (IQR 3.0–8.0 days), and rectal (IQR 3.0–7.0 days) swabs and 4 days for urine (*n* = 10; IQR 2.5–7.0 days) (Figure 3A). Most of the samples were collected during the first week post symptoms onset. Within this period, 74.1% (*n* = 453) of lesion swabs, 74.0% (*n* = 259) of oropharyngeal swabs, 87.2% (*n* = 41) of rectal swabs, and 90.0% (*n* = 9) of urine tested positive for MPXV. In samples collected from 8 until 14 days following symptoms onset, 21.9% (*n* = 134) of lesion swabs, 22.0% (*n* = 77) of oropharyngeal swabs, 12.8% (*n* = 6) of rectal swabs and 10.0% (*n* = 1) of urine were positive. In samples collected more than 15 days after symptoms onset, 2.6% (*n* = 16) of lesion swabs and 3.1% (*n* = 11) of oropharyngeal swabs were positive.

Regarding the evolution of MPXV loads, a constant median *C*_t_ value was obtained over the days after the onset of symptoms in lesions swabs (median *C*_t_ = 23 with a minor variation of ±1–2 cycles). However, a considerable viral load decrease was observed in oropharyngeal swabs after 15 days from the onset of symptoms, and in rectal samples after 8 days from the onset of symptoms (Figure 3B–E). The low number of urines did not allow taking any robust conclusion.

Of note, the few lesion and oropharyngeal swabs collected more than 21 days from the onset of symptoms were from two hospitalized patients with severe mpox disease and HIV‐positive status, showing the difficulty for viral clearance.

**Figure 4:**
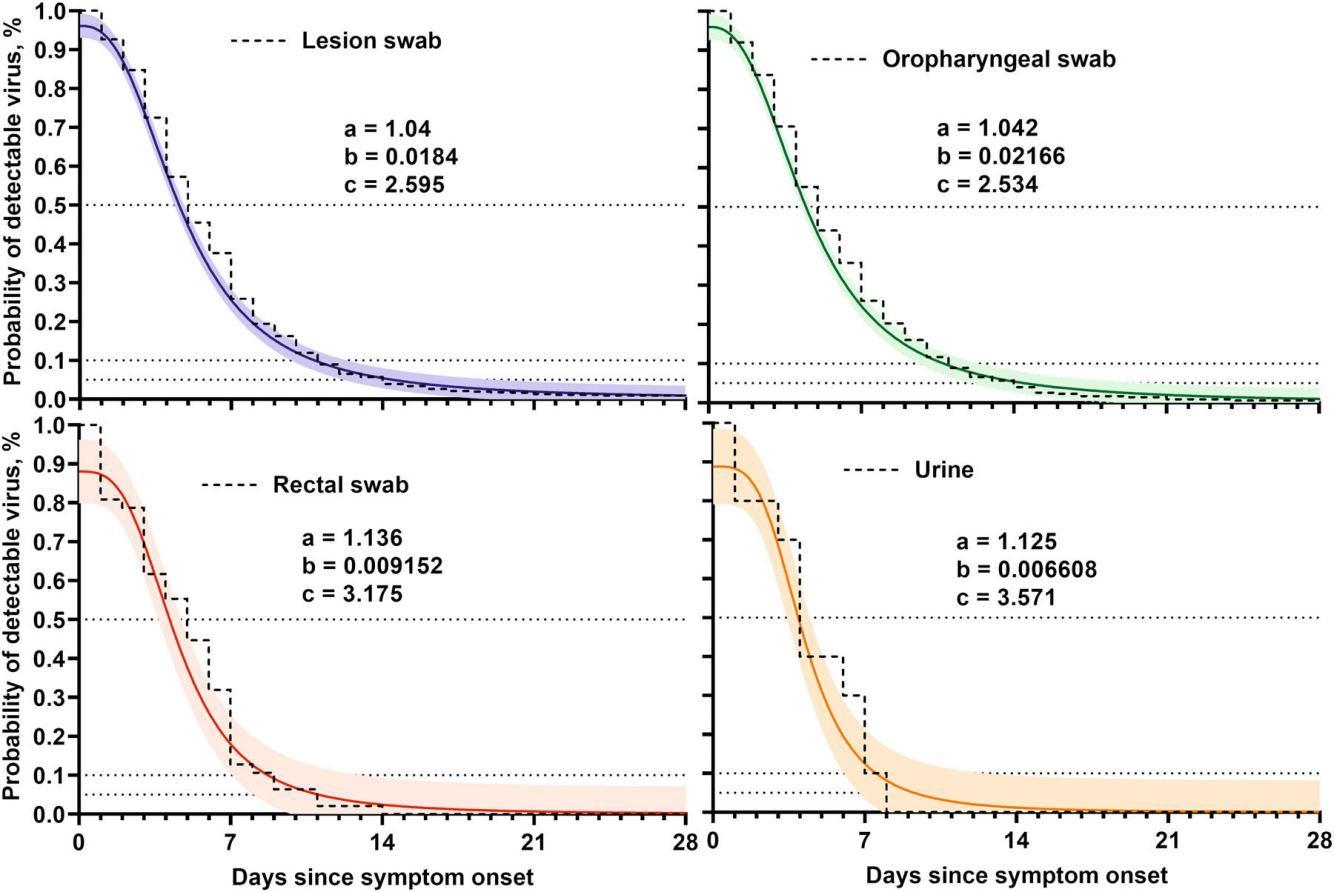
Time to viral clearance in all patients. The dashed line represents the cumulative incidence calculated on the observed data. Parameters *a*, *b*, and *c* for each type of sample are shown.

In parallel, another analysis was also performed with the same set of samples (611 lesion swabs, 352 oropharyngeal swabs, 47 rectal swabs and 10 urines), to observe the decreasing evolution of viral load over the days of symptoms (Figure 4). A 95% confidence interval with the Farazdaghi‐Harris density model was determinate, and a coefficient of determination *R* ^2^ > 0.97 was calculated. The median time obtained for 50% of patients confirms the values obtained in the previous analysis: 5 days for lesions (95% CI, IQR 4–5), oropharyngeal (95% CI, IQR 4–5), and rectal (95% CI, IQR 3–5) swabs and 4 days for urine (95% CI, IQR 3–5). According to the model, 90% of patients would have undetectable viral DNA in lesions and oropharyngeal swabs in 11 days (95% CI, IQR 10–12) after symptoms onset. The corresponding estimates for other samples were 9 days (95% CI, IQR 7–13) in rectal swabs and 8 days (95% CI, IQR 6–12) in urine samples. Time to viral clearance for 95% of patients was 15 days (95% CI, IQR 12–19) for lesions and oropharyngeal swabs, 11 days (95% CI, IQR 8–inf) for rectal swabs and 9 days (95% CI, IQR 7–inf) for urine samples (Figure 4 and Table S4).

However, the results obtained for urine samples must be confirmed with a higher number of samples, to provide robustness to these observations.

### 3.4 Evolution of the MPXV Viral Load Throughout the Infection Period Since Symptoms Onset

**Figure 5:**
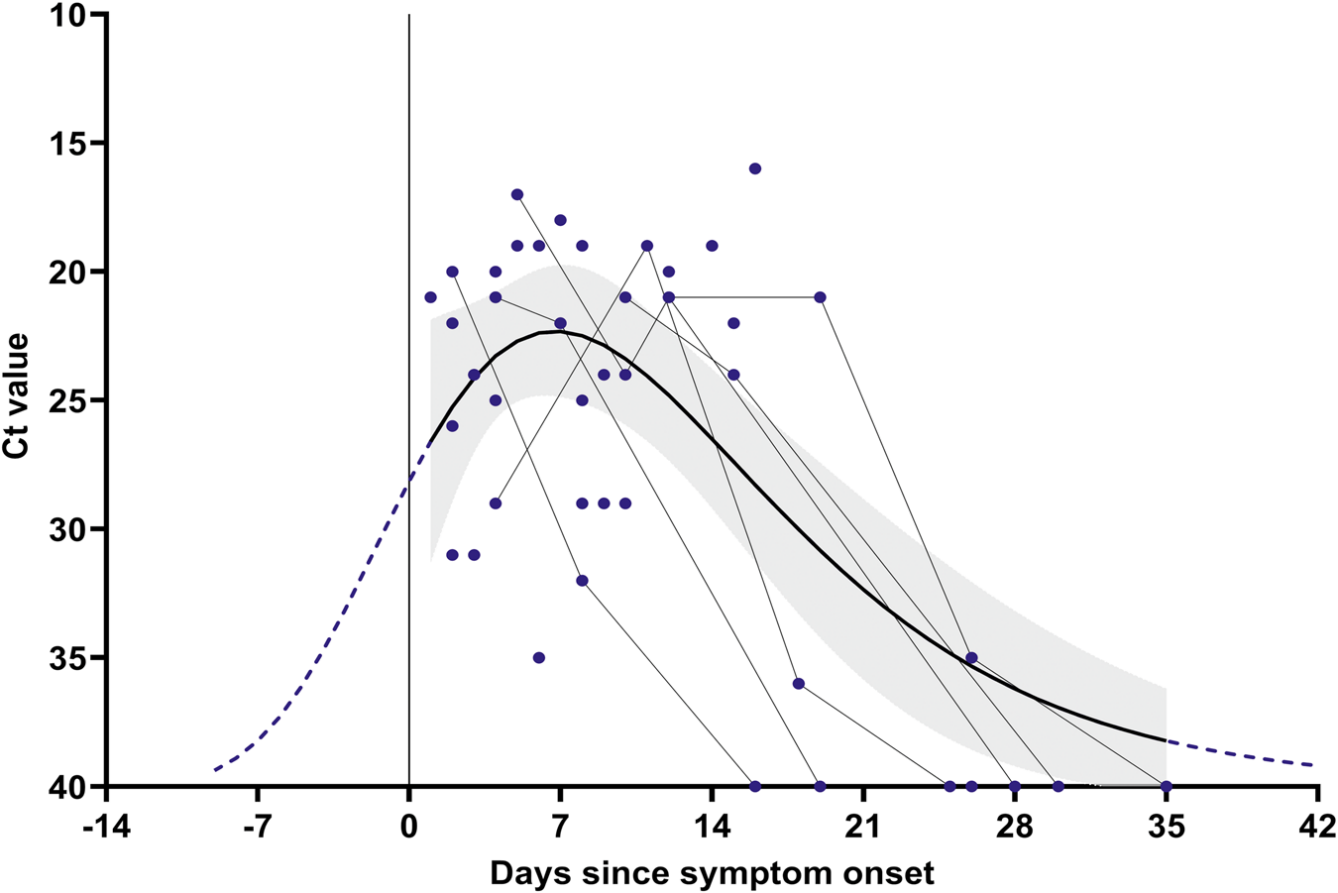
PCR *C*_t_ values for MPXV in several lesion swabs (≥ two samples) from the same patient throughout the infection period since symptoms onset (Day 1) (*n* = 19). Grey lines connect samples from the same patient with three or more lesion swab samples (*n* = 6). The black continuous line estimates the time course of infection for visualization. The blue dashed line projects the curve before and after available data. The grey shadow indicates a 95% confidence interval. *C*_t_ values are represented in the *C*_t_ axis inverted for better interpretation purposes.

To describe the evolution of viral load over the days since symptoms onset until the end of infection, we analysed the *C*_t_ values of samples collected from 19 patients with at least two positive swabs samples collected on different days and for whom data on the onset of signs were available. The *C*_t_ results of 47 lesion swab samples were evaluated using an adaptation of the Gompertz growth curve [10]. Using the least squares method, the parameters *a* = 0.1154, *b* = 416.6, and *c* = 6.747 were found with a coefficient of determination of *R* ^2^ = 0.45. The *C* parameter represents the day of the highest viral load and equals 6.747 days, indicating that the peak viral load was reached after approximately 7 days, with a mean *C*_t_ value of 22 (Figure 5).

Within this data set, 14 patients (*n* = 14/19; 73.7%) had the first positive result in the first week (≤ 7 days) after the symptom's onset and the remaining five in the second week (8–14 days) (*n* = 5/19; 26.3%). At 14 days after symptoms onset, seven patients (*n* = 7/19; 36.8%) presented lesion samples negative by PCR (*C*_t_ ≥ 40) (Figure 5).

Overall, the viral load showed an initial increase (illustrated by a decline in the PCR *C*_t_ values), followed by a substantial decrease (illustrated by an escalation of *C*_t_ values). Across patients, a constant median *C*_t_ value was observed in samples collected on ≤ 7 days (*n* = 20; median *C*_t_ = 22), 8–14 days (*n* = 14; median *C*_t_ = 23), and 15–21 days (*n* = 7; median *C*_t_ = 24) since symptoms onset, decreasing considerably after 21 days (*n* = 6; median *C*_t_ = 40) (Table S5).

According to the extrapolation carried out, the incubation period of MPXV infection obtained was 7 days before symptoms onset.

### 3.5 MPXV Viral Load of Paired Samples From Different Types Collected From the Same Patient

**Figure 6:**
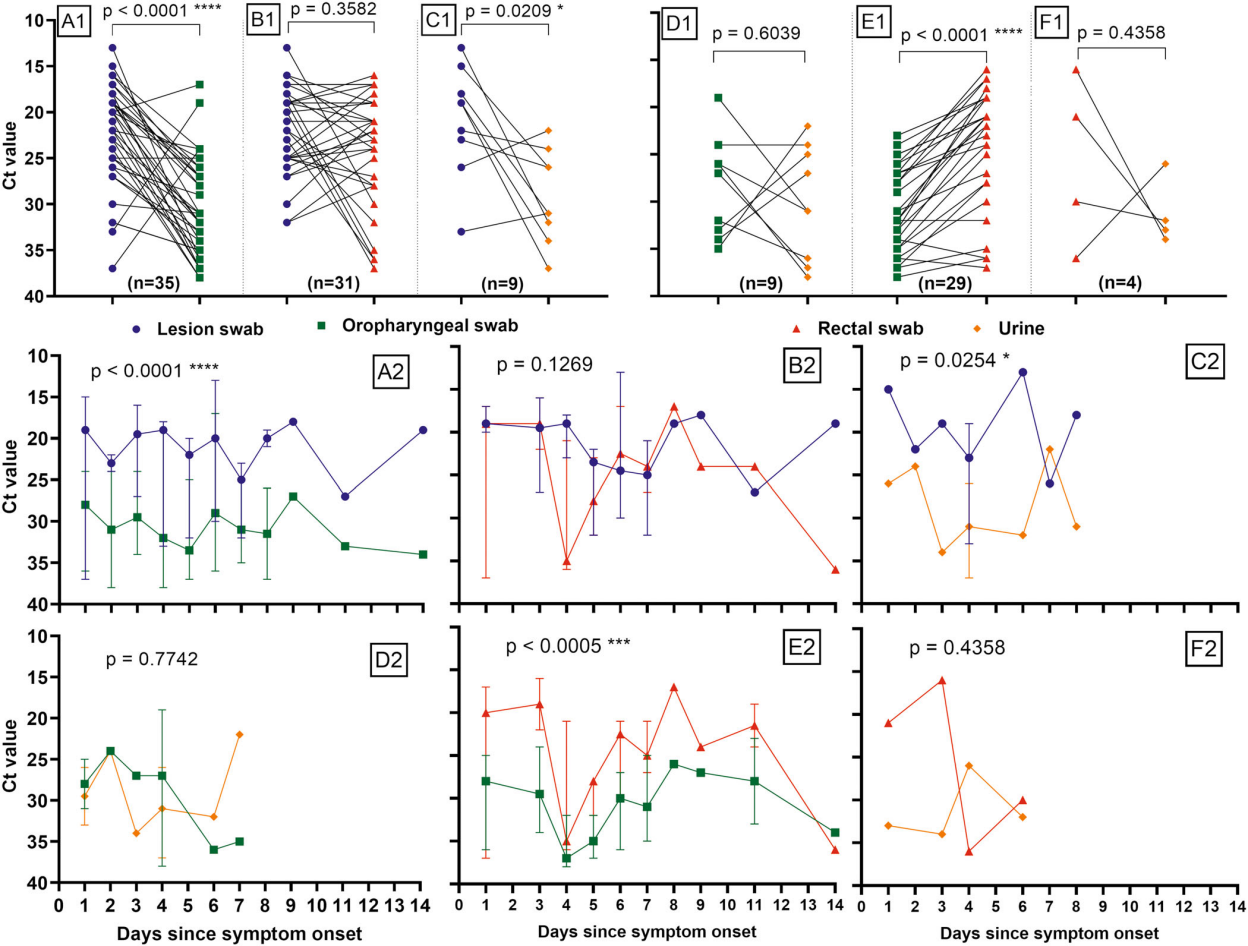
MPXV median *C*_t_ values of paired samples from different types, collected on the same day from the same patient, throughout the infection period since symptoms onset. Markers indicate PCR *C*_t_ values of lesion swabs (blue dots), oropharyngeal swabs (green squares), rectal swabs (red triangles) and urine samples (orange hexagons). (A1–F1) Black lines link PCR *C*_t_ values from different types of samples collected on the same day from the same patient. (A2)–(F2) represents the data of the (A1)–(F1) panels throughout the infection period since symptoms onset. Median *C*_t_ values with the upper and lower limits are displayed. The *y*‐axis scale is inverted for better interpretation purposes, as lower *C*_t_ values represent higher viral loads. *p* values are accompanied by asterisks, depending on the degree of significance (i.e., from one asterisk for *p* values less than 0.05 to four asterisks for *p* values less than 0.0001).

MPXV viral loads were compared between paired samples from different types collected on the same day from the same patient: lesion versus oropharyngeal swabs (*n* = 35), lesion versus rectal swabs (*n* = 31), lesion swabs versus urine (*n* = 9), oropharyngeal versus rectal swabs (*n* = 29); oropharyngeal swabs versus urine (*n* = 9), and rectal swabs versus urine (*n* = 4) (Figure 6A1–F1). The median *C*_t_ level in different samples over time was also compared (Figure 6A2–F2). As expected, the results mirrored the ones obtained when analysing all positive samples from all 953 patients.

Briefly, lesion swabs presented statistically significant lower median *C*_t_ values when compared to oropharyngeal swabs (*C*_t_ = 21 vs. *C*_t_ = 31; *p* < 0.0001) and urine (*C*_t_ = 19 vs. *C*_t_ = 31; *p* = 0.0209), indicating higher viral loads (Figure 6A1,C1, Table S6), whereas no statistically significant differences were found when compared with rectal swabs (*C*_t_ = 22 vs. *C*_t_ = 23; *p* = 0.3582) (Figure 6B1, Table S6). Likewise, rectal swabs presented lower median *C*_t_ values when compared to oropharyngeal swabs (*C*_t_ = 22 vs. *C*_t_ = 31; *p* < 0.0001) and urine (*C*_t_ = 25 vs. *C*_t_ = 32; *p* = 0.4358) (Figure 6E1,F1, Table S6). In addition, no statistically significant differences were found when comparing oropharyngeal swabs with urines (*C*_t_ = 27 vs. *C*_t_ = 31; *p* = 0.6039) (Figure 6D1, Table S6).

Regarding the viral load between different types of samples over the days since symptoms onset, a similar pattern was observed where statistical relationships were maintained considering these data throughout the days of symptoms (Figure 6A2–F2).

## 4 Discussion

In Portugal, the mpox epidemic pattern mirrored the ones of other non‐endemic countries, with the first cases detected throughout May and the epidemic peak being reached in June 2022. We detected the first cases on May 17, 2022 [7], but a retrospective study performed with samples from the beginning of the year confirmed that the virus was already circulating in Portugal, before the first cases were notified. Patients were mostly MSM in the 30–39 age group. Most of the positive cases were of foreign origin and were identified in all regions of the country, with the Lisbon Metropolitan Area showing the highest number of cases.

Previous genomic studies identified subclade IIb (B.1 sublineages) in all MPXV genomes sequenced in Portugal in the study period [14].

Current epidemiologic and surveillance information indicates that mpox is now a globally distributed infectious disease that can be spread from person to person based on close skin‐to‐skin contact without zoonotic exposure. The emergence of this disease has been characterized by a rapid international spread with a growing number of cases in new geographic areas, a preponderance of disease among members of a distinct social group, and a somewhat atypical clinical presentation, collectively being shaped in part by increased international travel, human behaviour, and viral pathogenicity.

In this study, 4125 samples from 2016 patients with suspected MPXV infection were evaluated. MPXV was detected in 1702 samples from 953 patients from multiple sites. We took advantage of the availability of a high number of heterogeneous samples from multiple patients to establish different study approaches: (i) overall assessment of MPXV PCR positivity in different clinical sample types; (ii) assessment of the evolution of the MPXV viral load throughout the infection period, from symptoms onset to viral clearance; and (iii) assessment of MPXV viral load of different types of paired samples collected from the same patient.

The use of other sample types may prove useful to the diagnosis of mpox cases. In fact, some of the patients included in our study did not have lesions at the time of diagnosis, and about 5% (*n* = 38) of patients were diagnosed through oropharyngeal swabs while having negative lesions, suggesting that this sample type may also constitute a complimentary approach for this purpose. In the recent outbreak, the clinical presentation of the disease did not always include a disseminated rash and the presence of the virus has already been reported in the oropharynx [15, 16, 17, 18, 19]. The oropharynx can be a source of transmission through oral contact (e.g., kissing) and saliva exchange. So, it is important to diagnose the infection at the beginning of the prodromal phase, avoiding contagions before the presentation of other manifestations of the disease [20, 21].

Although the accuracy of urine samples to diagnose mpox seems to be considerably lower when compared with other analysed sample types, its detection rate of about 41% in the current data set shows that it should not be discarded and, similarly to oropharyngeal swabs, can constitute a complimentary diagnostic approach, on behalf of a mpox outbreak with the above‐cited characteristics. Other studies have shown the inconsistency of MPXV detection in urine samples [15]. Nevertheless, urine was clearly the least represented sample type in our data set (*n* = 34 from the 953 positive patients), so a higher number of urine samples is needed to take more robust conclusions.

Most of the samples were collected during the first week post symptoms onset. The median time from symptoms onset was 5 days for lesions, oropharyngeal and rectal swabs. Within the first 7 days following symptoms onset, most samples, regardless of type, tested PCR positive for MPXV. Nevertheless, a considerable viral load decrease was observed after 15 days from the onset of symptoms in oropharyngeal swabs and after 8 days in rectal swabs. In contrast, a constant median *C*_t_ value was obtained until about 30 days after the onset of symptoms in lesions swabs. These data not only reinforces the higher accuracy of lesion swabs to diagnose mpox when compared with other sample types but also suggest that the lesions, in patients of this specific outbreak, can be frequent in multiple stages of the disease development. This contrasts with what has been described in mpox cases in endemic countries [4, 22]. Despite the apparent maintenance of a high viral load for a long period in lesion swabs, these results show the importance of performing the diagnosis within 15 days of the onset of symptoms, enhancing the probability of detecting the virus. Severe cases of mpox may constitute an exception, as it is believed that the virus may remain in the patient's body for months [12]. The model we used to access viral clearance also showed that the lesion and oropharyngeal swabs had the longest median time to viral clearance (15 days) from symptoms onset, followed by rectal swabs (11 days), somehow supporting the results described above. Of note, in another study the authors obtained much higher viral clearance times [23]. However, a direct comparison between these studies cannot be straightforwardly performed because, although the other study enrolled fewer patients and samples, it was able to evaluate more samples per patient and over a longer period of time, which likely ensured more robust data.

A time course of infection was extrapolated with samples from patients who had multiple lesion swabs collected on different days. We observed that, in general, the incubation period of MPXV infection was 7 days before symptoms onset, the peak viral load was reached approximately 7 days after symptoms onset, and after 14 days, the MPXV viral load decreased. These data are in line with what has already been reported in other studies, in which mpox infection usually resolves 2–4 weeks after symptoms onset [24, 25].

Among the future challenges, we would highlight that studies should be conducted to explore the association between viral load and a patient's symptomatology and clinical signs. In our case, without access to clinical data, it was not possible to conduct such an analysis. In addition, considering the expansion of clade Ib in African countries and its potential dissemination in non‐endemic countries, we believe that it would be important to carry out the same study of sample adequacy for diagnostic, in samples with clade Ib strains.

Globally, these results suggest that the most suitable samples for detecting MPXV in an outbreak with the above‐described specificities are the lesions and rectal swabs. As only the lesions seem to maintain a robust viral load for several weeks, sample collection carried out within the first 7 days after the onset of symptoms is recommended. These findings, together with the observation that some patients did not display lesions and others (about 5%) were diagnosed through oropharyngeal swabs while having negative lesions, suggest that, whenever possible, multisite testing should be performed to increase MPVX detection efficiency.

## Author Contributions

Conceptualization: Rita Cordeiro and Fernando da Conceição Batista. Resources and data curation: Rita Cordeiro, Fernando da Conceição Batista, Nuno Verdasca, and Cláudia Palhinhas. Writing–original draft preparation: Rita Cordeiro and Fernando da Conceição Batista. Writing–review and editing: João Paulo Gomes. Coordination of laboratory activities: Rita Cordeiro, Ana Pelerito, Isabel Lopes de Carvalho, and Maria Sofia Núncio. Collection and processing of clinical samples: Rita Cordeiro, Ana Pelerito, Isabel Lopes de Carvalho, Sílvia Lopo, Raquel Neves, Raquel Rocha, Paula Palminha, Maria José Borrego, Carla Manita, Idalina Ferreira, Célia Bettencourt, Patricia Vieira, Sónia Silva, Ivone Água‐Doce, Carla Roque, Dora Cordeiro, Greice Brondani, João Almeida Santos, Susana Martins, Irene Rodrigues, and Carlos Ribeiro. Supervision: João Paulo Gomes and Maria Sofia Núncio. All authors have read and agreed to the published version of the manuscript.

## Conflicts of Interest

The authors declare no conflicts of interest.

## Supporting information

Supporting information.

## Data Availability

The data that support the findings of this study are available on request from the corresponding author. The data are not publicly available due to privacy or ethical restrictions.
