# Supplementary material for: An Overview of Monkeypox Virus Detection in Different Clinical Samples and Analysis of Temporal Viral Load Dynamics
Source: J Med Virol. 2024 Dec 9;96(12):e70104. doi: 10.1002/jmv.70104 (PMC11628931; doi:10.1002/jmv.70104)
Supplement: Supplementary file 1 — Supporting information. [file JMV-96-e70104-s001.docx]

**Supplemental material**

**Table S1**: Number of analysed samples and confirmed cases between May 17, 2022 and May 24, 2023.

| **Analysis date** | **Analysed samples** | **Positive samples*** | **Confirmed cases** |
| --- | --- | --- | --- |
| **May/22** | 259 | 152 | 118 |
| **Jun/22** | 974 | 513 | 297 |
| **Jul/22** | 866 | 463 | 256 |
| **Aug/22** | 957 | 386 | 202 |
| **Sep/22** | 450 | 106 | 58 |
| **Oct/22** | 210 | 38 | 16 |
| **Nov/22** | 88 | 6 | 1 |
| **Dec/22** | 105 | 14 | 2 |
| **Jan/23** | 50 | 5 | 1 |
| **Fev/23** | 30 | 3 | 0 |
| **Mar/23** | 46 | 10 | 2 |
| **Apr/23** | 50 | 4 | 0 |
| **May/23** | 40 | 2 | 0 |
| **Total** | **4125** | **1702** | **953** |

* The number of positive samples is considerably higher than the number of confirmed cases due to the existence of multiple samples for some patients and the need for follow-up in some cases.

**Table S2:** Confirmed cases by age group and gender.

| **Age group** | **Confirmed cases (%)** | **Gender** | |
| --- | --- | --- | --- |
|  |  | **Female (%)** | **Male (%)** |
| **0-9** | 0 | 0 | 0 |
| **10-19** | 5 (0.5) | 0 | 5 (0.5) |
| **20-29** | 278 (29.2) | 2 (22.2) | 276 (29.2) |
| **30-39** | **420 (44.1)** | 7 (77.8) | 413 (43.8) |
| **40-49** | 187 (19.6) | 0 | 187 (19.8) |
| **50-59** | 55 (5.8) | 0 | 55 (5.8) |
| **60+** | 8 (0.8) | 0 | 8 (0.8) |
| **Total (%)** | **953** | **9 (0.9)** | **944 (99.1)** |

**Table S3:** Confirmed cases by country region and information of resident population and population density.

| **Portugal NUTS*** | **Confirmed cases (%)** | **Total resident population^1^ (%)** | **Population density^2^ (Nº/km^2^)** |
| --- | --- | --- | --- |
| **North Region** | 158 (16.6) | 3 620 740 (34.7) | 170.6 |
| **Central Region** | 27 (2.8) | 2 254 545 (21.6) | 80.0 |
| **Lisbon Metropolitan Area** | **742 (77.9)** | 2 891 658 (27.7) | 961.7 |
| **Alentejo Region** | 5 (0.5) | 713 964 (6.8) | 22.6 |
| **Algarve Region** | 17 (1.8) | 470 992 (4.5) | 94.5 |
| **Autonomous Region of Azores** | 1 (0.1) | 239 368 (2.3) | 103.3 |
| **Autonomous Region of Madeira** | 3 (0.3) | 252 976 (2.4) | 316.1 |
| **Total** | **953** | **10 444 242** | **113.5** |

*****The Nomenclature of Territorial Units for Statistics (NUTS) is developed by Eurostat, and is employed in both Portugal and the entire European Union for statistical purposes. The NUTS branch extends from NUTS1, NUTS2 and NUTS3 regions, with the complementary Local Administrative Units (LAU) sub-categorization being used to differentiate the local areas, of trans-national importance. NUT 2 categorization of Portugal regions was used.

1. Instituto Nacional de Estatística, “Estimativas anuais da população residente – Densidade populacional por Local de residência,” accessed November 17, 2023, [https://www.ine.pt/xportal/xmain?xpid=INE&xpgid=ine_main](https://www.ine.pt/xportal/xmain?xpid=INE%26xpgid=ine_main).
2. PORDATA Estatísticas sobre Portugal e Europa, “Base de dados Portugal Contemporâneo,” accessed November 17, 2023, <https://www.pordata.pt/>.

**Table S4**: Time to viral clearance in 50%, 90% and 95% patients.

| **Sample type** | **Time (days) to viral clearance in 50% of patients**  **(95% CI)** | **Time (days) to viral clearance in 90% of patients**  **(95% CI)** | **Time (days) to viral clearance in 95% of patients**  **(95% CI)** |
| --- | --- | --- | --- |
| **Lesion swab** | 5 (4-5) | 11 (10-12) | 15 (12-19) |
| **Oropharyngeal swab** | 5 (4-5) | 11 (10-12) | 15 (12-19) |
| **Rectal swab** | 5 (3-5) | 9 (7-13) | 11 (8-inf) |
| **Urine** | 4 (3-5) | 8 (6-12) | 9 (7-inf) |

**Table S5**: Number of lesion swabs and patients included in evolution analysis of viral load in the period since symptom onset until the end of infection.

| **Days since symptom onset** | **Median Ct value** | **Lesion swab** | **Patients** |
| --- | --- | --- | --- |
| **≤7 days** | 22 | 20 | 14 |
| **8-14 days** | 23 | 14 | 12 |
| **15-21 days** | 24 | 7 | 7 |
| **22-28 days** | 40 | 4 | 4 |
| **>28 days** | 40 | 2 | 2 |

**Table S6**: MPXV median Ct values of paired samples from different types (see Figure 6 for more details).

| **Median Ct value** | | | | **p value** | **Figure** |
| --- | --- | --- | --- | --- | --- |
| **Lesion swab** | **Oropharyngeal swab** | **Rectal swab** | **Urine** |  |  |
| 21 | 31 | NA | NA | p<0.0001 | **A1** |
| 22 | NA | 23 | NA | p=0.3582 | **B1** |
| 19 | NA | NA | 31 | p=0.0209 | **C1** |
| NA | 27 | NA | 31 | p=0.6039 | **D1** |
| NA | 31 | 22 | NA | p<0.0001 | **E1** |
| NA | NA | 25 | 32 | p=0.4358 | **F1** |

NA – Not applicable.
